# Evaluation of Mechanical Properties of Different Thermoplastic Orthodontic Retainer Materials after Thermoforming and Thermocycling

**DOI:** 10.3390/polym15071610

**Published:** 2023-03-23

**Authors:** Alaa T. Albilali, Bashayer H. Baras, Mohammad A. Aldosari

**Affiliations:** 1 Department of Pediatric Dentistry and Orthodontics, College of Dentistry, King Saud University, Riyadh 11545, Saudi Arabia; maldosari@ksu.edu.sa; 2 Department of Restorative Dental Sciences, College of Dentistry, King Saud University, Riyadh 11545, Saudi Arabia; bbaras@ksu.edu.sa

**Keywords:** orthodontic thermoplastic retainers, retainer materials, thermoforming, thermocycling, mechanical properties, flexural modulus, hardness, roughness

## Abstract

While the durability of thermoplastic aligners has been the subject of numerous studies, the durability of thermoplastic retainers has received significantly less attention. Patients are often advised to wear their thermoplastic retainers indefinitely, so the durability of the materials used in their fabrication is crucial to determining whether they are worth the cost. Limited studies have evaluated the properties of thermoplastic retainer materials and the effects of thermocycling on their mechanical properties. Thus, this study aimed to examine six thermoplastic retainer materials after thermoforming with and without thermocycling. The materials’ flexural modulus, hardness, and surface roughness values were measured after thermoforming (Group 1) and after thermoforming with subsequent thermocycling for 10,000 cycles (Group 2). After thermoforming, there was a significant difference in flexural modulus and hardness values between most of the materials. However, their surface roughness was not significantly different (*p* < 0.05). After thermocycling, the flexural modulus and hardness increased significantly for most tested materials (*p* < 0.05) compared to Group 1. Concerning the surface roughness, only two materials showed significantly higher values after thermocycling than Group 1. Thus, all the mechanical properties of the evaluated materials differed after thermoforming, except the surface roughness. Moreover, while thermocycling made the materials stiffer and harder in general, it also made some of them rougher.

## 1. Introduction

Retention is necessary to keep teeth corrected and prevent relapse after orthodontic treatment [1]. It also improves long-term patient satisfaction after such treatments [2]. However, a previous study that evaluated several orthodontic patients found that teeth tend to relapse to their pretreatment condition, even in the case of treatments with ideal results [3].

Owing to the lack of selection criteria, the choice of the retainer is usually governed by the preferences of the patient and clinician [4]. Most patients favor thermoplastic retainers because of their aesthetics and invisible appearance. Thermoplastic retainers are removable orthodontic devices made of different polymeric materials. They are more economical and easier to make than conventional acrylic and wire-based retainers [5]. Thus, thermoplastic retainers are used widely, and it has been reported that their use is associated with high patient satisfaction and compliance [6,7].

The fabrication of thermoplastic retainers involves heating during vacuum or pressure forming, which can permanently change the material’s morphology and thermal properties. Moreover, during their placement and removal from the oral cavity, thermoplastic materials are subjected to mechanical, thermal, and chemical degradation, which manifests at their morphological level and can deteriorate their properties [8,9]. As a result, their efficiency in maintaining the position of the teeth is compromised. Moreover, the clinical use of thermoplastic retainers has several limitations. For instance, they can undergo fracturing and exhibit short life spans, cracking, discoloration, wear, and water absorption [10,11,12].

Generally, retention protocols vary widely among practitioners, and there is no consensus regarding the optimal protocol [13]. However, there is now broad acceptance of the need for lifelong retention. Several researchers have suggested that long-term retention is the only way to ensure stability [1,14,15,16]. Nevertheless, the long-term use of thermoplastic retainers in the oral cavity tends to degrade the retainer material over time [8]. Additionally, the common drawbacks of thermoplastic retainers are their low survival rate and frequent need for replacement [5,17,18]. Therefore, stronger and more durable materials should be used to fabricate thermoplastic retainers to reduce costs and sustain orthodontic therapy outcomes.

The physical properties of the different aligner materials available have been studied extensively. Previous studies have found that thermoforming and increasing the environmental temperature degrade the mechanical properties of thermoplastic aligner materials [19,20,21]. However, another study found that thermocycling did not have a significant effect on the thermomechanical properties of a few aligner materials (Duran and Erkodur^®^) [22].

There have been a few studies on the physical properties of thermoplastic retainer materials. For example, a previous study investigated the effects of thermoforming and intraoral use on the mechanical properties of a PETG retainer material [23]. They evaluated the surface roughness, compressive strength, and tensile strength and found that intraoral use increased the compressive strength, tensile strength, elastic modulus, and surface roughness of the material. In addition, previous studies evaluated the effect of different cleaning methods on Vivera, Essix ACE, and Essix C+ retainers’ physical properties, including the roughness, light transmittance, and flexural modulus [24,25,26].

Although many studies have evaluated the durability of thermoplastic aligner materials, only a few have analyzed the durability of thermoplastic retainer materials. Because thermoplastic retainers are prescribed for life, the durability of such materials is a determining factor of their cost-effectiveness.

To the best of our knowledge, no study has elucidated the effects of in vitro aging (thermocycling) on the physical properties of the following thermoplastic retainer materials in order to predict their clinical usefulness [27]: Zendura, Essix Plus, Tru-Tain DX, Duran Plus, Essix ACE, and Imprelon^®^ S pd. Moreover, no previous study has compared their flexural modulus, hardness, and surface roughness values.

Therefore, in this study, we evaluated the effects of thermocycling on the mechanical properties of the six above-mentioned thermoplastic retainer materials. Specifically, we measured their mechanical properties (flexural modulus, hardness, and surface roughness) after thermoforming and after thermoforming with subsequent thermocycling. The null hypotheses of the study were as follows:

(1) There are no differences in the mechanical properties of the investigated thermoplastic retainer materials after thermoforming.
(2) There are no differences in the mechanical properties of the investigated thermoplastic retainer materials after thermoforming and subsequent thermocycling.
(3) Thermocycling does not have a statistically significant effect on the mechanical properties of the investigated thermoplastic retainer materials.

## 2. Materials and Methods

Six different thermoplastic retainer materials were assessed (as mentioned in Table 1). Since a thickness of 1 mm is preferred by most clinicians [28], we used samples of this thickness in the current study. To evaluate the mechanical properties of the six thermoplastic retainer materials, we divided the test samples into two groups as follows:

Group 1: samples subjected to thermoforming.

Group 2: samples subjected to thermoforming and thermocycling.

**Table 1:** Thermoplastic retainer materials analyzed in this study (their compositions were obtained from their material safety data sheets (MSDSs)).

Material Name	Manufacturer	Composition
Zendura item #9169	Bay Materials LLC, Fremont, CA, USA	Polyurethane (PU)
Essix Plus	Dentsply Raintree Essix, Bradenton, FL, USA	Copolyester
Tru-Tain DX	Tru-Tain, MN, USA	Copolyester
Duran Plus	Scheu-Dental GmbH, Iserlohn, Germany	PETG Copolyester
Essix ACE	Dentsply Raintree Essix, Bradenton, FL, USA	PET Copolyester
Imprelon S pd	Scheu-Dental GmbH, Iserlohn, Germany	Copolyester

### 2.1. Thermoforming

**Figure 1:**
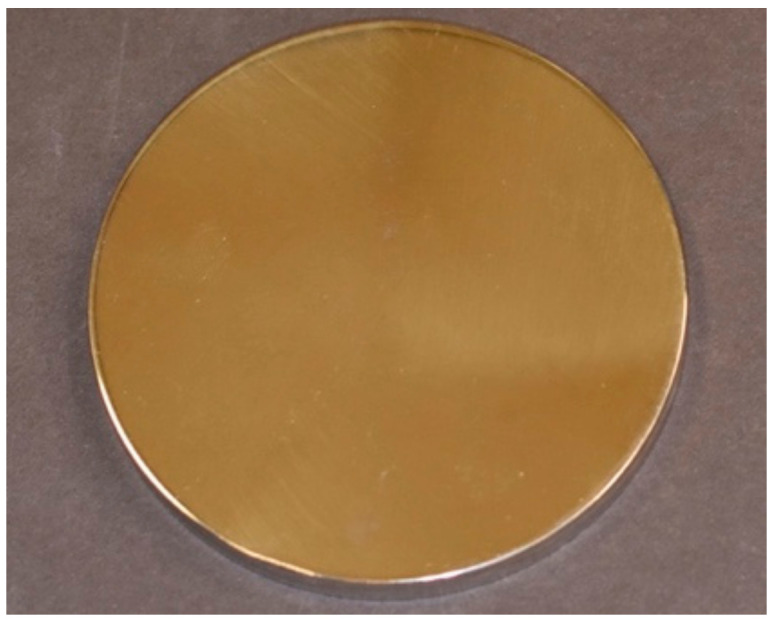
Stainless-steel plate (110 × 10 mm^2^).

**Figure 2:**
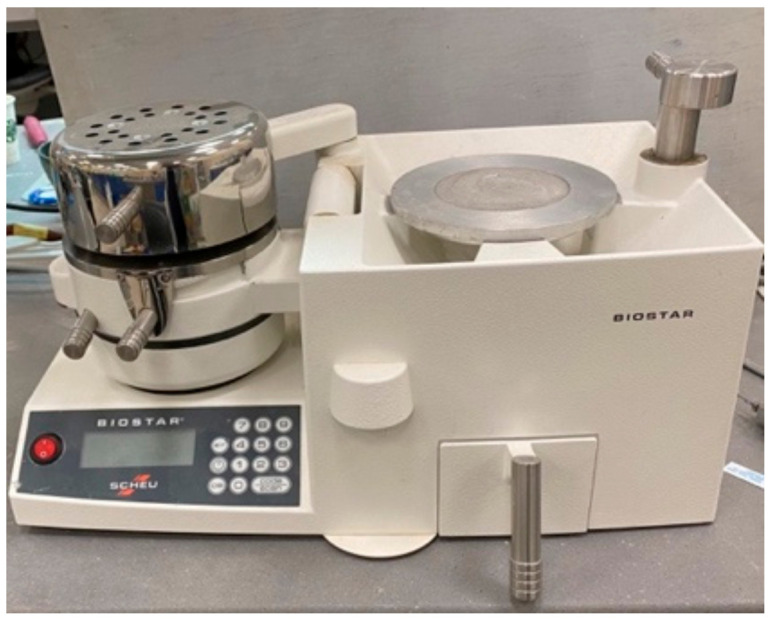
BioStar VI vacuum forming machine.

Sheets of the thermoplastic retainer materials were first heated and subjected to a vacuum as per the manufacturer’s instructions over a stainless-steel plate with a 110 mm diameter and 10 mm height (Figure 1). Thermoforming was performed using a BioStar VI vacuum forming machine (Scheu-Dental GmbH, Iserlohn, Germany) (Figure 2).

### 2.2. Thermocycling

**Figure 3:**
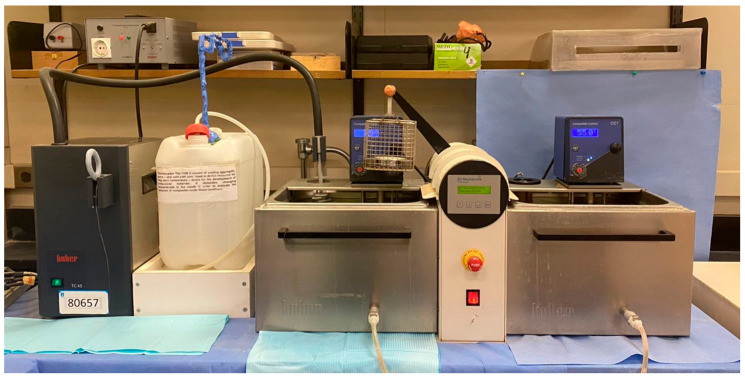
Thermocycler 1100 (SD-Mechatronik, Westerham, Germany).

To compare the six thermoplastic retainer materials in terms of their durability, we subjected them to thermocycling after thermoforming (Group 2). This study subjected the samples to 10,000 thermocycles utilizing the thermocycler 1100 (SD-Mechatronik, Westerham, Germany) (Figure 3) to simulate one year of intraoral use. Samples were immersed in 37 °C distilled water prior to thermocycling for 24 h. The dwelling time in the water at 5 and 55 °C was 15 s, and the dripping time was 10 s [27].

### 2.3. Flexural Modulus

#### 2.3.1. Specimen Preparation

Thermoplastic sheets were formed, as mentioned in Section 2.1. After the thermoforming process, specimens with dimensions of 40 × 10 mm^2^ were cut using scissors, and their edges were finished using a polishing machine (Jean Wirtz, Dusseldorf, Germany) [29].

#### 2.3.2. Evaluation of Flexural Modulus

**Figure 4:**
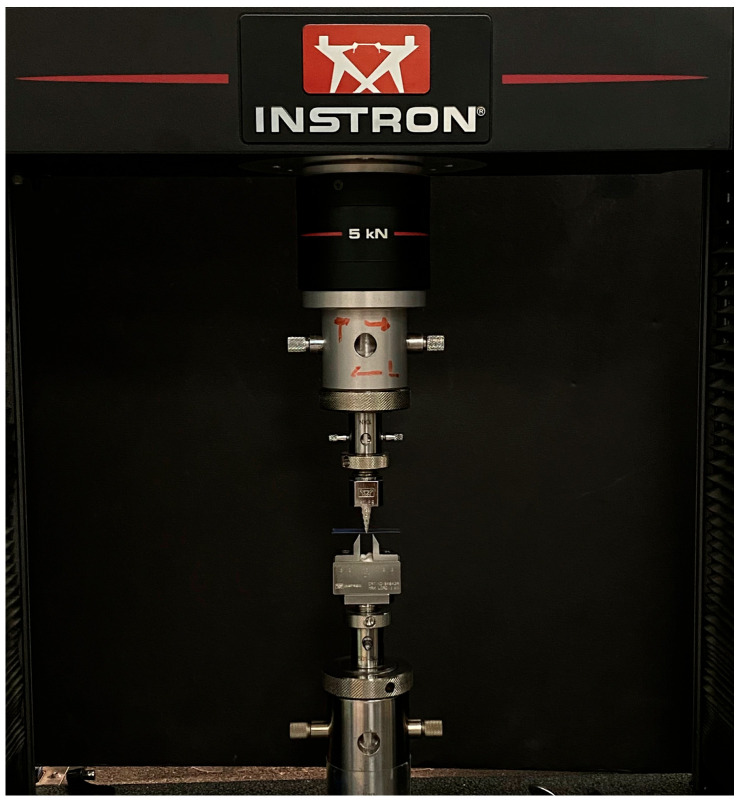
Three-point testing using Instron 5965 system (Instron, Canton, MA, USA).

The three-point bending test was performed at a span length of 8 mm [29] using an Instron 5965 system (Instron, Canton, MA, USA) (Figure 4) to calculate the flexural modulus. Specimens were loaded to a maximum deflection of 5 mm and at a speed of 1 mm/min [22]. The flexural modulus was calculated in giga pascal (GPa) using the following equation:

$$E=\frac{F_{1}l^{3}}{4bh^{3}d}$$

where *F*_1_ is the highest load in the straight-line section of the load–deflection curve, *d* is the magnitude of the deflection at *F*_1_, *l* is the span length between the supports, *b* is the width of the test sample, and *h* is its height.

### 2.4. Hardness

#### 2.4.1. Specimen Preparation

Thermoplastic sheets were formed, as mentioned in Section 2.1. After the thermoforming process, specimens with dimensions of 10 × 10 mm^2^ were cut using scissors. Their edges were polished with a polishing machine (Jean Wirtz, Dusseldorf, Germany), using water as the coolant.

#### 2.4.2. Evaluation of Hardness

**Figure 5:**
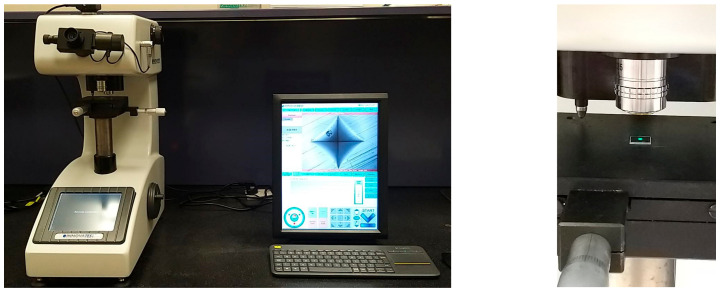
Hardness testing using Innovatest hardness testing machine (Nova 130, Maastricht, Netherlands).

Instrumented indentation was performed using a hardness testing machine (Nova 130, Innovatest, Maastricht, The Netherlands) (Figure 5) with a Vickers indenter. The Vickers hardness was determined by applying a force of 10 N for 10 s.

(1) $$HV=1.854\frac{F}{d^{2}}$$

where *F* is the force applied on the surface, and *d* is the average diameter of the indentations.

### 2.5. Surface Roughness

#### 2.5.1. Specimen Preparation

Thermoplastic sheets were prepared as described in Section 2.1. Next, samples with dimensions of 10 × 10 mm^2^ were prepared and assigned to Groups 1 and 2.

#### 2.5.2. Evaluation of Surface Roughness

**Figure 6:**
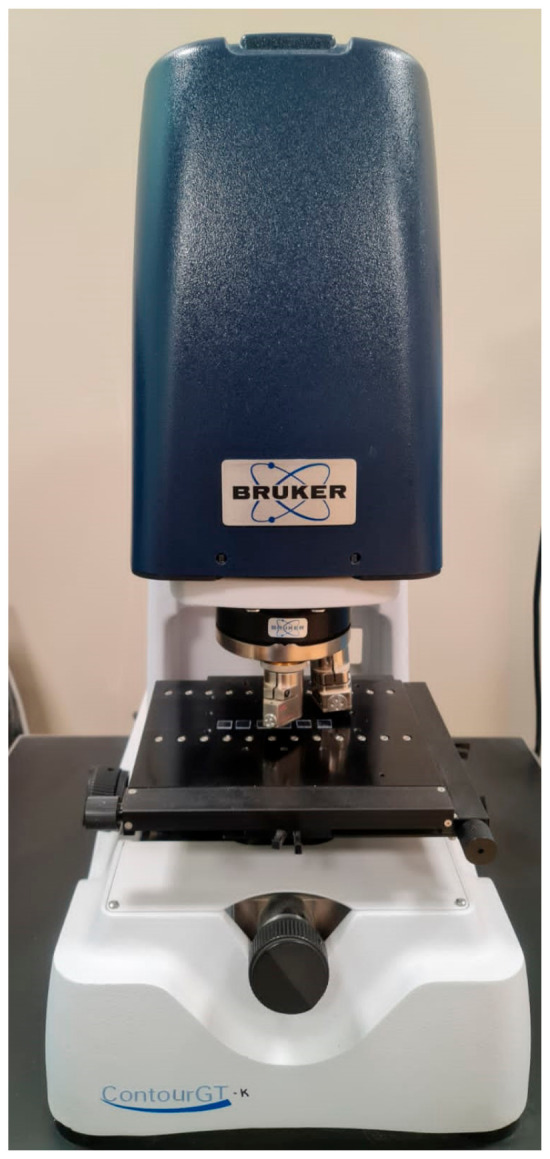
Surface roughness evaluation using Bruker Contour GTK (Bruker nano GmbH, Berlin, Germany).

A Bruker Contour GTK (Bruker Nano GmbH, Berlin, Germany) optical noncontact surface profiling system (Figure 6) was used to measure the surface roughness. This system utilizes noncontact scanning white-light interferometry for 3D surface configuration. The average roughness value (R_a_) of each specimen was calculated. Three tracings were performed on each specimen at three different locations (2 mm from each other along a predetermined line across the specimen). The mean of these values was taken as the surface roughness value. The data were processed using the Vision 64 software (Bruker Nano GmbH, Berlin, Germany) accompanying the Bruker Contour GT-K system [30].

### 2.6. Statistical Analysis

For the flexural modulus and hardness measurements, at α = 0.05, with a power of 85%, the total sample size was determined to be at least 60 specimens. Thus, 72 specimens were prepared and used for the flexural strength and hardness evaluations. Specifically, 12 specimens of each thermoplastic retainer material were prepared. For the surface roughness evaluations, at α = 0.05, with a power of 90%, the total sample size was also determined to be at least 60 specimens. In this case, 72 specimens (12 of each material) were prepared and used to evaluate the surface roughness.

The obtained data were analyzed using SPSS^®^ Version 23 (SPSS Inc., IBM, Chicago, IL, USA). The flexural strength, hardness, and surface roughness data were tested for normality using the Shapiro–Wilk test. After the conditions of normality had been satisfied, one-way analysis of variance (ANOVA) was performed at a significance level of *p* < 0.05, along with Tukey’s honestly significant difference (HSD) post hoc test for multiple comparisons. Subsequently, the Mann–Whitney U test was used to check for significant differences between Group 1 and Group 2.

## 3. Results

### 3.1. Mechanical Properties of Thermoplastic Retainer Materials after Thermoforming (Group 1)

#### 3.1.1. Flexural Modulus

The flexural modulus of Tru-Tain DX was the highest (2.35 ± 0.26 GPa), followed by those of Zendura (2.32 ± 0.55 GPa), Duran Plus (2.30 ± 0.18 GPa), Essix ACE (2.09 ± 0.13 GPa), Essix Plus (1.78 ± 0.23 GPa), and Imprelon S pd (1.76 ± 0.45 GPa) (see Table 2).

The results of one-way ANOVA indicated statistically significant differences between the flexural modulus values of the various materials (*p* < 0.05) (Table 2). Tukey’s HSD post hoc test for multiple comparisons showed statistically significant differences in the flexural modulus values between Imprelon S pd and Zendura, Imprelon S pd and Duran Plus, Imprelon S pd and Tru-Tain DX, Zendura and Essix Plus, Duran Plus and Essix Plus, and Essix Plus and Tru-Tain DX (*p* < 0.05) (see Table 2).

#### 3.1.2. Hardness

The Vickers hardness number of Zendura was the highest (12.70 ± 0.11), followed by those of Duran Plus (10.37 ± 0.15), Tru-Tain DX (9.15 ± 0.05), Essix ACE (9.10 ± 0.06), Essix Plus (9.08 ± 0.04), and Imprelon S pd (8.82 ± 0.08) (see Table 3).

The results of one-way ANOVA indicated statistically significant differences in the hardness values of the various materials (*p* < 0.05) (see Table 3). Tukey’s HSD post hoc test for multiple comparisons showed statistically significant differences in the hardness values of all the materials (*p* < 0.05), except between Essix Plus and Essix ACE, Essix Plus and Tru-Tain DX, and Essix ACE and Tru-Tain DX (*p* > 0.05) (see Table 3).

#### 3.1.3. Roughness

The roughness of Zendura was the highest (0.28 ± 0.30 µm), followed by those of Essix ACE (0.11± 0.07 µm), Essix Plus (0.08 ± 0.03 µm), Imprelon S pd (0.07 ± 0.01 µm), Duran Plus (0.06 ± 0.03 µm), and Tru-Tain DX (0.06 ± 0.01 µm) (see Table 4).

The results of one-way ANOVA indicated that there were no statistically significant differences in the roughness values of the various materials (*p* > 0.05) (see Table 4).

### 3.2. Mechanical Properties of Thermoplastic Retainer Materials after Thermocycling (Group 2)

#### 3.2.1. Flexural Modulus

The flexural modulus of Zendura was the highest (2.85 ± 0.28 GPa), followed by those of Duran Plus (2.56 ± 0.22 GPa), Essix ACE (2.36± 0.18 GPa), Tru-Tain DX (2.30 ± 0.18 GPa), Imprelon S pd (2.05 ± 0.19 GPa), and Essix Plus (1.96 ± 0.19 GPa) (see Table 5).

The results of one-way ANOVA showed statistically significant differences in the flexural modulus values of the various materials (*p* < 0.05). Tukey’s HSD post hoc test for multiple comparisons showed statistically significant differences in the flexural modulus values of all the materials (*p* < 0.05), except between Imprelon S pd and Essix Plus, Duran Plus and Essix ACE, and Essix ACE and Tru-Tain DX (*p* > 0.05) (see Table 5).

#### 3.2.2. Hardness

The Vickers hardness value of Zendura was the highest (13.13 ± 0.12), followed by those of Duran Plus (11.40 ± 0.13), Essix ACE (10.30 ± 0.14), Tru-Tain DX (10.25 ± 0.14), Essix Plus (9.55 ± 0.21), and Imprelon S pd (9.38 ± 0.04) (see Table 6).

The results of one-way ANOVA showed statistically significant differences in the hardness values of the various materials (*p* < 0.05) (see Table 6). Tukey’s HSD post hoc test for multiple comparisons showed statistically significant differences in the hardness values of all the materials (*p* < 0.05), except between Imprelon S pd and Essix Plus and between Essix ACE and Tru-Tain DX (*p* > 0.05) (see Table 6).

#### 3.2.3. Roughness

The roughness value of Zendura was the highest (1.08 ± 0.25 µm), followed by those of Essix Plus (0.15 ± 0.13 µm), Essix ACE (0.13 ± 0.02 µm), Tru-Tain DX (0.09± 0.04 µm), Imprelon S pd (0.09 ± 0.05 µm), and Duran Plus (0.07 ± 0.02 µm) (see Table 7).

The results of one-way ANOVA showed statistically significant differences in the surface roughness of the evaluated materials (*p* < 0.05) (Table 7). Tukey’s HSD post hoc test for multiple comparisons showed statistically significant differences in the roughness between Imprelon S pd and Zendura, Zendura and Duran Plus, Zendura and Essix Plus, Zendura and Essix ACE, and Zendura and Tru-Tain DX (*p* < 0.05) (Table 7).

### 3.3. Effects of Thermocycling on Mechanical Properties of Thermoplastic Retainer Materials

#### 3.3.1. Flexural Modulus

**Figure 7:**
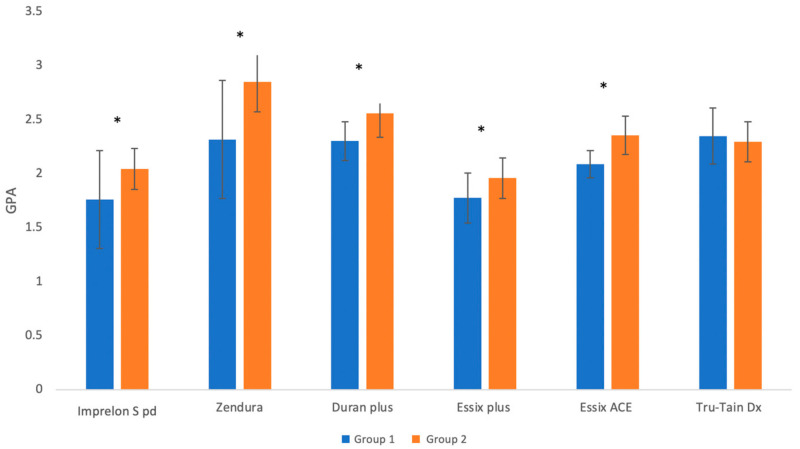
Mean ± SD of flexural modulus values of Group 1 and Group 2 (the * symbol indicates a significant difference between the two groups for the same material at *p* < 0.05 level).

The mean difference in the flexural modulus values of Group 1 and Group 2 was the highest in the case of Zendura, followed by Imprelon S pd, Essix ACE, Duran Plus, and Essix Plus. The mean flexural modulus of Tru-Tain DX was slightly higher than that before aging (difference not significant). The Mann–Whitney U test showed that the mean (SD) flexural modulus was significantly higher after aging for all the materials (*p* < 0.05) except Tru-Tain DX (*p* > 0.05) (Figure 7).

#### 3.3.2. Hardness

**Figure 8:**
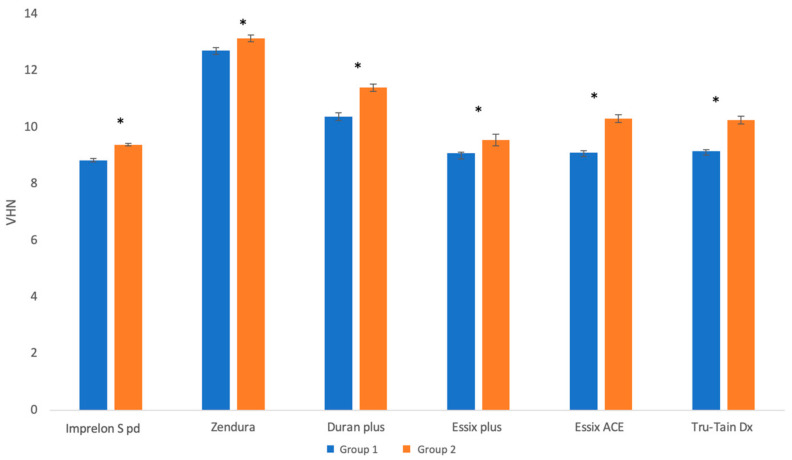
Mean ± SD of hardness values of Group 1 and Group 2 (the * symbol indicates a significant difference between the two groups for the same material at *p* < 0.05 level).

The mean difference in the hardness values of Group 1 and Group 2 was the highest for Essix ACE, followed by Tru-Tain DX, Duran Plus, Imprelon S pd, Essix Plus, and Zendura. The Mann–Whitney U test showed that the mean (SD) hardness was significantly higher after aging for all the materials (*p* < 0.05) (Figure 8).

#### 3.3.3. Surface Roughness

**Figure 9:**
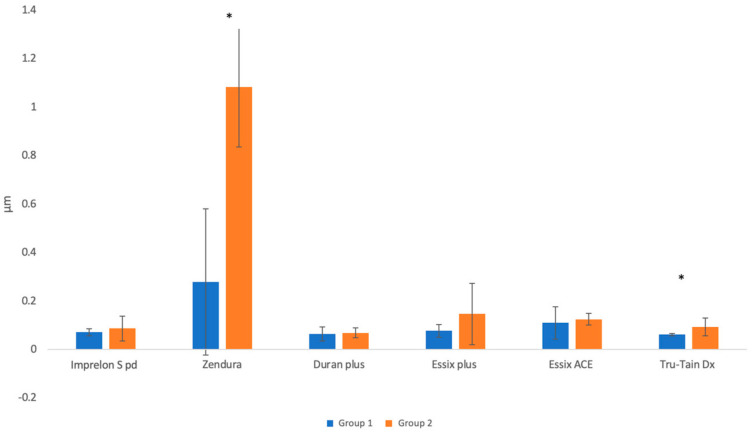
Mean ± SD of surface roughness values of Group 1 and Group 2 (the * symbol indicates a significant difference between the two groups for the same material at *p* < 0.05 level).

The mean difference after aging was the highest in the case of Zendura, followed by Essix Plus, Tru-Tain DX, Imprelon S pd, Essix ACE, and Duran Plus. The Mann–Whitney U test showed that the mean (SD) surface roughness was significantly higher after aging only in the cases of Zendura and Tru-Tain DX (*p* < 0.05) (Figure 9).

## 4. Discussion

There is now broad acceptance of the need for lifelong retention after orthodontic treatments [1,14,15]. Therefore, reliable and cost-effective retainers have become essential. In this study, the three-point bending test, hardness, and surface roughness measurements were performed to evaluate the mechanical properties of six thermoplastic orthodontic retainer materials after subjecting them to thermoforming and thermoforming with subsequent thermocycling. Thermocycling was performed to identify the materials that exhibited the best mechanical properties after in vitro aging to reduce the cost and sustain the outcomes of orthodontic therapy.

The six retainer materials’ mechanical properties were compared after being subjected to thermoforming (Group 1) and thermoforming with subsequent thermocycling (Group 2). After thermoforming, there were significant differences in the properties between the retainer materials. Imprelon S Pd and Essix Plus showed significantly lower flexural modulus values than Tru-Tain DX, Zendura, and Duran Plus. However, there were no significant differences in the flexural modulus between Tru-Tain DX, Zendura, Duran Plus, and Essix ACE. After thermoforming and thermocycling, there were significant differences in the flexural modulus between all the materials, except between Imprelon S pd and Essix Plus, Essix ACE and Duran Plus, and Essix ACE and Tru-Tain DX. After thermocycling, all materials’ flexural moduli increased significantly, except for True-Tain Dx.

The Vickers hardness values of the six materials were measured after thermoforming and thermocycling. After thermoforming, there were significant differences between all the materials, except between Essix Plus and Essix ACE, Essix Plus and Tru-Tain DX, and Essix ACE and Tru-Tain DX. After thermoforming and thermocycling, there were statistically significant differences in the hardness values between all the materials, except between Imprelon S Pd and Essix Plus and between Essix ACE, and Tru-Tain DX. The hardness values of all the materials increased significantly after thermocycling.

The surface roughnesses of the six retainer materials were evaluated after thermoforming and after thermoforming and subsequent thermocycling. After thermoforming alone (Group 1), there were no statistically significant differences between the materials. However, after thermoforming and thermocycling (Group 2), Zendura showed significantly higher surface roughness than other materials. Additionally, thermocycling significantly increased the surface roughnesses of Zendura and Tru-Tain DX compared to their roughness values in Group 1.

Therefore, all the null hypotheses were rejected except for the first null hypothesis in terms of surface roughness.

Thermocycling has been used in previous studies to evaluate the effects of aging on orthodontic thermoplastic materials. Most studies on thermoplastic aligner materials performed 100–2500 thermocycles. However, in the current study, since we aimed to evaluate the expected lifespan of orthodontic thermoplastic retainer materials, we performed 10,000 thermocycles to simulate one year of intraoral use [27]. While previous studies have provided valuable insights into the mechanical properties of different aligner and retainer thermoplastic materials, it is difficult to compare their findings, as they used a wide range of experimental designs and methods [19,22,24,25,26,29,31,32]. Moreover, no ISO specifications or national standards have been proposed for the measurement of the mechanical properties of thermoplastic orthodontic materials. Several test standards, such as ISO 20795-2 for orthodontic base polymers [33], have been published and implemented in the literature. However, these standards tend to emphasize stiffer orthodontic materials that exhibit more uniform material stresses and have greater thicknesses, such as polymethyl methacrylate (PMMA) used to fabricate Hawley retainers [29].

Kwon, Lee [31] evaluated the effects of thermocycling (1000 cycles between 5 °C and 55 °C for a dwelling time of 15 s) on the mechanical properties of thermoplastic aligner materials (Essix A+, Essix C+, and Essix ACE). After being subjected to thermocycling, the hardness values of the thermoplastic aligner materials increased, as was also observed in the present study. However, the delivered force decreased after thermocycling. A previous study [34] evaluated the effects of thermocycling on the mechanical properties of thermoplastic aligner materials (Duran, Hardcast, and polyurethane). They subjected the aligner materials to thermocycling for 500 and 2500 cycles (between 5 and 55 °C). After 500 cycles, the hardness values of the materials were not significantly affected, whereas their elastic modulus values decreased dramatically. However, after 2500 thermal cycles, the hardness and elastic modulus values of most of the thermoplastics decreased dramatically, in contrast to the current study’s findings, wherein most of the materials showed an increase in the hardness and flexural modulus values after thermocycling. A recent study [22] evaluated the flexural modulus and hardness of PTEG aligner materials (Duran and Erkodur) after thermocycling (200 cycles between 5 °C and 55 °C for 20 s). They found that the flexural modulus increased slightly after thermocycling. However, the increase was not statistically significant. This could be due to the low number of cycles used. In their study, thermocycling significantly increased the hardness of Erkodur but had little effect on the hardness of Duran [22].

Regarding the reasons behind choosing three-point bending in the current study, the primary benefit of three-point bending is attributed to the simplicity of the sample geometry. So, establishing a setup is easy and relatively inexpensive relative to other mechanical properties. In addition, because the fundamental mechanism is well understood and the number of influential variables is limited, the analysis of the findings is relatively straightforward [29]. Moreover, three-point bending could represent the forces applied to the thermoplastic retainer during placement in and removal from the oral cavity, clenching, and bruxism. A span length of 8 mm was used in the current study based on the recommendation of previous studies [29,32]

As properties, the flexural modulus and hardness are frequently confused. Both characteristics capture how a material behaves in response to contact. The hardness reflects a material’s resistance to penetration, whereas the flexural modulus indicates a material’s resistance to bending (stiffness or rigidity). Suitable thermoplastic materials should exhibit high wear resistance (high hardness), low surface roughness, and a flexural modulus of at least 1500 MPa as per ISO 20795-2:2013 [33]. In this study, we evaluated the effects of in vitro aging on the properties of different thermoplastic retainer materials. After in vitro aging, all the materials exhibited good flexural modulus values. The flexural modulus of all the materials increased significantly, except for that of Tru-Tain DX, which showed no significant changes. In addition, the hardness values of all the materials increased significantly.

Moreover, the surface roughnesses of only Zendura and Tru-Tain DX increased with aging. However, previous research has shown that the roughness was detected by the tongue only if it was above 0.5 μm on average [35]. In the current study, only Zendura exhibited a roughness of more than 0.5 μm after thermocycling. Yet, a rough surface should be avoided, as it allows more plaque and bacteria to adhere, resulting in halitosis, carious lesions, and periodontal diseases [36].

It has been reported that thermoforming, water absorption, and temperature all impact the mechanical properties of thermoplastic materials [37]. In this study, after 10,000 thermal cycles, the flexural modulus and hardness values of the investigated materials (Group 2) were higher than their baseline values (Group 1).

High temperatures and wet environments result in polymer oxidation [38]. A few researchers have hypothesized that the increased flexural modulus or stiffness is attributable to the oxidation of the polymer [38]. The findings of this study are supported by previous observations that water hydrolysis causes physiochemical changes in polyurethanes, leading to their expansion and irreversible deterioration [39]. Moreover, it has been demonstrated that aging thermoplastic polyurethanes exhibit increased flexural moduli [40]. Thermoplastic copolyesters have been shown to be more wear-resistant than softer thermoplastics [41]. However, copolyesters have low hydrolytic stability [42]. Polyethylene polymers are susceptible to high temperatures (100–130 °C), which cause them to warp, bend, and deform [42].

In addition, differences in the molecular weight, chemical composition, density, additives, degree of polymerization, and crystallinity among the different types of thermoplastic retainers could explain the observed variations in the mechanical properties between different materials. Changes in the crystalline and amorphous structures of the thermoplastic materials or the release of plasticizers could account for the observed increases in the flexural modulus and hardness following thermocycling [43]. However, the actual reasons for these increases need to be elucidated in future studies. Future studies should perform Fourier transform infrared (FTIR) spectroscopy to identify the composition of each polymeric material. Additionally, the volume fractions of the amorphous and crystalline phases should be evaluated through X-ray diffraction analysis. FTIR and X-ray diffraction would help in explaining the reasons for the observed differences between materials and the increase in the mechanical properties (flexural modulus and hardness) after thermocycling.

The results of this study provide a glimpse into the durability of thermoplastic retainer materials. Nevertheless, the results of this study should be interpreted with caution, as clinical conditions differ sharply from the simulated oral conditions used in this study. Although the results of this study are not conclusive, they reveal how thermoplastic retainer materials act in a wet environment and how they age. However, this study has several limitations that must be considered. For instance, samples with a thickness of only 1 mm were used in this study. Future studies should compare samples with different thicknesses. The standardization of rectangular samples is a limitation. Retainers are clinically formed using a stone model that represents the patient’s teeth. Because this study did not evaluate retainers that were shaped like a patient’s dentition, differences that could have resulted from variations in the sample shapes may not have been accounted for. The duration of the thermocycling process is another limitation. Even though the samples were subjected to 10,000 thermocycles, the thermocycling time should be increased, as retainers are typically worn for much longer periods than one year. This study was able to elucidate the time-dependent changes in the mechanical properties of thermoplastic retainer materials. However, additional research is needed to yield more comprehensive results. Moreover, the tear strength, tensile strength, and creep of the materials should also be evaluated. In addition, clinical studies should be conducted to evaluate the effects of intraoral aging on the properties of the different materials used to fabricate thermoplastic retainers.

## 5. Conclusions

The cost-effectiveness of thermoplastic retainers is dependent on their durability, as they are typically prescribed for life. In order to predict their clinical performance, the current study attempted to evaluate the impact of in vitro aging (thermocycling) on the physical properties of different types of thermoplastic retainer materials. After thermoforming, there were significant differences in the flexural modulus and hardness values between most of the investigated thermoplastic retainer materials. However, the differences in the roughness values were insignificant between materials after thermoforming. In addition, thermocycling had a significant effect on the mechanical properties of the materials. In general, thermocycling made the retainer materials stiffer and harder and roughened a few of them. The data obtained in this study should help in the analysis of the stability and durability of the mechanical properties of thermoplastic orthodontic retainer materials.

## Figures and Tables

**Table 2:** Descriptive analysis of flexural modulus values of Group 1 (the same small letter indicates no significant difference between materials at *p* < 0.05 level).

Material	Mean	Std. Deviation	Std. Error	95% Confidence Interval for Mean		Min.	Max.
				Lower Bound	Upper Bound		
Imprelon S pd	1.76 ^a^	0.45	0.10	1.56	1.96	0.00	2.20
Zendura	2.32 ^b^	0.55	0.22	1.74	2.89	1.64	3.23
Duran Plus	2.30 ^b^	0.18	0.05	2.21	2.40	1.94	2.60
Essix Plus	1.78 ^a^	0.23	0.06	1.66	1.90	1.35	2.07
Essix ACE	2.09 ^a,b^	0.13	0.05	1.97	2.21	1.92	2.23
Tru-Tain DX	2.35 ^b^	0.26	0.11	2.08	2.62	2.04	2.80

**Table 3:** Descriptive analysis of hardness values of Group 1 (the same small letter indicates no significant difference between materials at *p* < 0.05 level).

Material	Mean	Std. Deviation	Std. Error	95% Confidence Interval for Mean		Min.	Max.
				Lower Bound	Upper Bound		
Imprelon S pd	8.82 ^a^	0.08	0.03	8.74	8.90	8.7	8.9
Zendura	12.70 ^b^	0.11	0.04	12.59	12.82	12.6	12.9
Duran Plus	10.37 ^c^	0.15	0.06	10.21	10.53	10.1	10.5
Essix Plus	9.08 ^d^	0.04	0.02	9.04	9.13	9.0	9.1
Essix ACE	9.10 ^d^	0.06	0.03	9.03	9.17	9.0	9.2
Tru-Tain DX	9.15 ^d^	0.05	0.02	9.09	9.21	9.1	9.2

**Table 4:** Descriptive analysis of roughness values of Group 1 (the same small letter indicates no significant difference between materials at *p* < 0.05 level).

Material	Mean	Std. Deviation	Std. Error	95% Confidence Interval for Mean		Min.	Max.
				Lower Bound	Upper Bound		
Imprelon S pd	0.07 ^a^	0.01	0.01	0.05	0.09	0.06	0.09
Zendura	0.28 ^a^	0.30	0.13	−0.09	0.65	0.05	0.69
Duran Plus	0.06 ^a^	0.03	0.01	0.03	0.10	0.05	0.12
Essix Plus	0.08 ^a^	0.03	0.01	0.05	0.11	0.06	0.12
Essix ACE	0.11 ^a^	0.07	0.03	0.03	0.19	0.07	0.23
Tru-Tain DX	0.06 ^a^	0.01	0.00	0.06	0.07	0.05	0.07

**Table 5:** Descriptive analysis of flexural modulus values of Group 2 (the same small letter indicates no significant difference between the materials at *p* < 0.05 level).

Material	Mean	Std. Deviation	Std. Error	95% Confidence Interval for Mean		Min.	Max.
				Lower Bound	Upper Bound		
Imprelon S pd	2.05 ^a^	0.19	0.05	1.94	2.16	1.72	2.42
Zendura	2.85 ^b^	0.28	0.07	2.69	3.01	2.36	3.27
Duran Plus	2.56 ^c^	0.22	0.06	2.42	2.70	2.26	2.88
Essix Plus	1.96 ^a^	0.19	0.05	1.85	2.07	1.62	2.34
Essix ACE	2.36 ^c,d^	0.18	0.05	2.25	2.46	2.06	2.68
Tru-Tain DX	2.30 ^d^	0.18	0.05	2.18	2.41	1.82	2.54

**Table 6:** Descriptive analysis of hardness values of Group 2 (the same small letter indicates no significant difference between the materials at *p* < 0.05 level).

Material	Mean	Std. Deviation	Std. Error	95% Confidence Interval for Mean		Min.	Max.
				Lower Bound	Upper Bound		
Imprelon S pd	9.38 ^a^	0.04	0.02	9.34	9.43	9.3	9.4
Zendura	13.13 ^b^	0.12	0.05	13.01	13.26	13.0	13.3
Duran Plus	11.40 ^c^	0.13	0.05	11.27	11.53	11.2	11.5
Essix Plus	9.55 ^a^	0.21	0.08	9.33	9.77	9.3	9.8
Essix ACE	10.30 ^d^	0.14	0.06	10.15	10.45	10.1	10.5
Tru-Tain DX	10.25 ^d^	0.14	0.06	10.11	10.40	10.1	10.5

**Table 7:** Descriptive analysis of roughness values of Group 2 (the same small letter indicates no significant difference between the materials at *p* < 0.05 level).

Material	Mean	Std. Deviation	Std. Error	95% Confidence Interval for Mean		Min.	Max.
				Lower Bound	Upper Bound		
Imprelon S pd	0.09 ^a^	0.05	0.02	0.02	0.15	0.06	0.18
Zendura	1.08 ^b^	0.25	0.11	0.78	1.39	0.80	1.48
Duran Plus	0.07 ^a^	0.02	0.01	0.04	0.09	0.05	0.10
Essix Plus	0.15 ^a^	0.13	0.06	−0.01	0.30	0.05	0.37
Essix ACE	0.13 ^a^	0.02	0.01	0.09	0.16	0.09	0.15
Tru-Tain DX	0.09 ^a^	0.04	0.02	0.05	0.14	0.06	0.15

## Data Availability

The data presented in this study are available on request from the corresponding author.
